# Investigating the role of phosphodiesterase Pde2 in coordinating resource reallocation through the environmental stress response

**DOI:** 10.1093/g3journal/jkag200

**Published:** 2026-09-22

**Authors:** Rachel A Kocik, Jamie Ahrens, Audrey P Gasch

**Affiliations:** Center for Genomic Science Innovation, University of Wisconsin-Madison, Madison, WI 53706, United States; Center for Genomic Science Innovation, University of Wisconsin-Madison, Madison, WI 53706, United States; Center for Genomic Science Innovation, University of Wisconsin-Madison, Madison, WI 53706, United States; Department of Medical Genetics, University of Wisconsin-Madison, Madison, WI 53706, United States

**Keywords:** Pde2, environmental stress, resource allocation, regulatory dynamics, fungi

## Abstract

Yeast responding to acute stress reallocates cellular resources, in part via the environmental stress response (ESR) that induces stress-defense genes while repressing ribosome biogenesis and growth genes. The purpose and regulation of coordinated induction and repression are incompletely understood, but both responses are influenced by ESR transcription factors Msn2 and Msn4 (Msn2/4). Here, we used single-cell microscopy and transcriptomic analysis to investigate the role of the upstream regulator Pde2 in ESR regulation and poststress fitness. Loss of *PDE2* weakened and shortened Msn2 activation following salt stress and produced muted induction of Msn2/4 targets, similar to a *msn2Δmsn4Δ* strain. In contrast, Pde2 had at most a minor impact on the ESR repressor Dot6, yet was important for repression of its targets beyond Msn2/4 influence. Consistent with our recent resource-reallocation model, *pde2Δ* cells had normal or faster poststress growth rates, despite weaker activation of the ESR. We discuss implications for ESR regulation and function.

## Teaser

As yeast respond to stress, they mount the Environmental Stress Response (ESR), inducing stress defense genes while repressing ribosome-biogenesis and growth genes. Kocik *et al*. proposed that a role for ESR gene repression is to reallocate resources toward induced genes. Here, the authors expanded on recent work to test the involvement of upstream regulator Pde2, a PKA-regulating phosphodiesterase, in regulating the ESR and contributing to resource reallocation during stress. Loss of *PDE2* weakened Msn2 activation and induction of Msn2/4-dependent genes following stress and contributed to ESR gene repression in unexpected ways. This work contributes to our understanding of resource allocation during adaptive responses.

## Introduction

Mounting a rapid cellular response to stressful insults often requires reallocation of cellular resources away from growth and division and toward cellular defense. While organisms have evolved in regulatory strategies, a common theme is the transcriptional and/or translational upregulation of defense genes and proteins and the downregulation of others that compete for transcriptional and translational machinery (Ho and Gasch 2015; Kocik et al. 2026). We previously hypothesized that fast-growing budding yeast, *Saccharomyces cerevisiae*, accomplishes this reallocation in part through a common stress-activated transcriptional program called the environmental stress response (ESR) (Gasch et al. 2000). Upon acute environmental stress, cells coordinately induce ∼300 genes involved in various aspects of stress defense (iESR genes) while repressing ∼600 genes encoding ribosomal proteins (RP), ribosome biogenesis factors (RiBi), and other proteins required for rapid growth (collectively, rESR genes).

Coupling iESR induction with rESR repression may aid in resource reallocation. Cells lacking the RiBi repressors *DOT6* and paralog *TOD6* fail to properly repress RiBi genes during stress (Lippman and Broach 2009; Ho et al. 2018). Instead, overly abundant RiBi transcripts remain associated with ribosomes at the expense of iESR mRNAs that show delayed ribosome association and translation (Ho et al. 2018). iESR induction is not required to survive single-stress treatments, but it is critical for increased tolerance to subsequent stress exposures (Berry and Gasch 2008; Berry et al. 2011; Guan et al. 2012). Consequently, delayed production of iESR proteins in *dot6Δtod6Δ* cells results in delayed acquisition of secondary-stress tolerance (Kocik et al. 2026). But mounting the response comes at a cost: Cells lacking iESR inducers Msn2 and Msn4 (Msn2/4) fail to properly mount the iESR during stress but, in fact, grow *faster* than wild type after single-stress treatments. In contrast, cells lacking Dot6/Tod6 induce the iESR without concomitant RiBi repression and grow slower than wild type after single-stress treatments (Ho et al. 2018; Bergen et al. 2022; Kocik et al. 2026). We proposed that rESR repression helps to balance the cost of iESR induction. Consistent with this model, a quadruple mutant lacking all 4 regulators grows equal to or slightly faster than wild-type cells responding to stress.

Here, we extended our recent work by investigating the upstream regulator Pde2, which inhibits the protein kinase A (PKA) pathway implicated in both iESR and rESR regulation. Under optimal conditions, PKA directly phosphorylates Msn2/4 and Dot6/Tod6 to restrict them to the cytosol (Beck and Hall 1999; Garreau et al. 2000; Deminoff et al. 2006; Lippman and Broach 2009; Huber et al. 2011). Upon stress, PKA inhibition, along with positive effects of stress-activated pathways, promotes nuclear translocation of Msn2/4 and Dot6/Tod6 and transcriptional regulation of their targets. How PKA is suppressed during stress remains incompletely resolved, but 1 mechanism is through the allosteric regulator cAMP. High cAMP levels activate PKA by disrupting PKA association with the inhibitory subunit Bcy1 (Kocik and Gasch 2022). cAMP turnover is regulated by phosphodiesterases, including high-affinity Pde2 activated by stress (as opposed to low-affinity Pde1 with no evidence of a role in stress responses) (Nikawa et al. 1987; Ma et al. 1999; Hu et al. 2010). Deletion of *PDE2* does not recapitulate the salt-dependent repression of the PKA phospho-proteomic network; however, we previously showed that Pde2 physically interacts with several PKA-regulated transcription factors, including Msn2, although the functional importance of that interaction is unknown (MacGilvray et al. 2018). Although PKA can directly phosphorylate Dot6, how Pde2 affects Dot6 activity has not been studied previously. Here, we investigated Msn2 and Dot6 activation dynamics, iESR and rESR transcriptome changes, and poststress fitness in *pde2Δ* cells responding to salt and other stresses. These results expand our understanding of ESR activation and our perspective that ESR activation is not required to survive single-stress conditions.

## Materials and methods

### Strains and growth conditions

*S. cerevisiae* strains of the BY4741 background used in this study are listed in Table 1. Strains were grown in low fluorescent media (LFM) as previously described (0.17% yeast nitrogen base without ammonium sulfate, folic acid, or riboflavin; 0.5% ammonium sulfate; 0.2% complete amino acid supplement; and 2% glucose) (Bergen et al. 2022; Kocik et al. 2026). Strain AGY1577 (*pde1Δpde2Δ*) was generated by replacing *PDE1* in the BY4741 *pde2::KANMX* strain (Open Biosystems) with the hygromycin-MX cassette via homologous recombination and validated using diagnostic PCRs. Crosses were used to generate AGY1575 (AGY1577 × AGY5), AGY1676 (AGY670 × AGY2228), and AGY2240 (AGY1676 × AGY2232). Liquid cultures for growth-rate assessment were grown as described in Kocik et al. (2026). In brief, test tubes inoculated with overnight liquid cultures grown ∼12 h in LFM were grown to an optical density at 600 nm of ∼0.1 before measurements were taken every 15 min. Growth rates were calculated using an exponential fit of OD measurements during 75 to 225 min after addition of stress, including 0.7 M NaCl or 3% ethanol added directly to the medium or centrifugation and resuspension of cells in LFM media with a pH of either 7.5 or 2.75. Replicates were done on different days with paired wild-type and mutant cultures, enabling replicate-paired T-test analysis.

**Table 1.** Strains used in this study.

Strain name	Description	Source
AGY4	*MATa*, *his3Δ1*, *leu2Δ0*, *ura3Δ0*, *met15Δ0*	Open Biosystems
AGY5	*MATα*, *his3Δ1*, *leu2Δ0*, *ura3Δ0*, *lys2Δ0*	Open Biosystems
AGY670	*MATa*, *his3Δ1*, *leu2Δ0*, *ura3Δ0*, *met15Δ0*, *pde2::KAN*	Open Biosystems
AGY1328	*MATa*, *his3Δ1*, *leu2Δ0*, *ura3Δ0*, *met15Δ0*, *DOT6-GFP(S65T)-His3MX*, *MSN2-mCherry-HYGMX*	Bergen et al. 2022
AGY1576	*MATa*, *his3Δ1*, *leu2Δ0*, *ura3Δ0*, *met15Δ0*, *pde1::HYG*	This study
AGY1577	*MATa*, *his3Δ1*, *leu2Δ0*, *ura3Δ0*, *met15Δ0*, *pde1::HYG*, *pde2::KAN*	This study
AGY1676	*MATa*, *his3Δ1*, *leu2Δ0*, *ura3Δ0*, *met15Δ0*, *pde2::KAN*, *DOT6-GFP(S65T)-His3MX*, *MSN2-mCherry-HYGMX*	This study
AGY2228	*MATα*, *his3Δ1*, *leu2Δ0*, *ura3Δ0*, *met15Δ0*, *DOT6-GFP(S65T)-His3MX*, *MSN2-mCherry-HYGMX*	Kocik et al. 2026
AGY2232	*MATα*, *his3Δ1*, *leu2Δ0*, *ura3Δ0*, *met15Δ0*, *NHP6A-iRFP*	Kocik et al. 2026
AGY2233	*MATa*, *his3Δ1*, *leu2Δ0*, *ura3Δ0*, *met15Δ0 DOT6-GFP(S65T)-His3MX*, *MSN2-mCherry-HYGMX*, *NHP6A-iRFP*	Kocik et al. 2026
AGY2240	*MATa*, *his3Δ1*, *leu2Δ0*, *ura3Δ0*, *pde2::KAN*, *DOT6-GFP(S65T)-His3MX*, *MSN2-mCherry-HYGMX*, *NHP6A-iRFP*	This study

### Microscopy and image analysis

Time-lapse microscopy was conducted using an FCS2 chamber (Bioptechs Inc., Butler, Pennsylvania) with image collection and analyses performed as previously described (Kocik et al. 2026). Msn2-mCherry and Dot6-GFP phenotypes were also determined as previously described, including “nuclear/cytoplasmic ratio” (defined as the average intensity of the top 5% of pixels divided by the median of all pixels) and acute stress peak height [defined as the maximum nuclear localization score immediately following NaCl addition {T13 to T20} minus the minimum score just before NaCl was added {T11 to T13}] (Bergen et al. 2022). Msn2 and Dot6 abundance were measured based on the median Msn2-mCherry or Dot6-GFP signal in each cell at each timepoint. The average signal of each cell before (T1 to T12) and after (T20 to T36) NaCl addition was plotted in the figures. Hierarchical clustering of cells in Fig. 1b and f was based on the log_2_(fold difference) compared to the population median. Clustering was performed using Gene Cluster 3.0 (Eisen et al. 1998) and visualized using Java TreeView version 1.2 (Saldanha 2004).

**Fig. 1.**
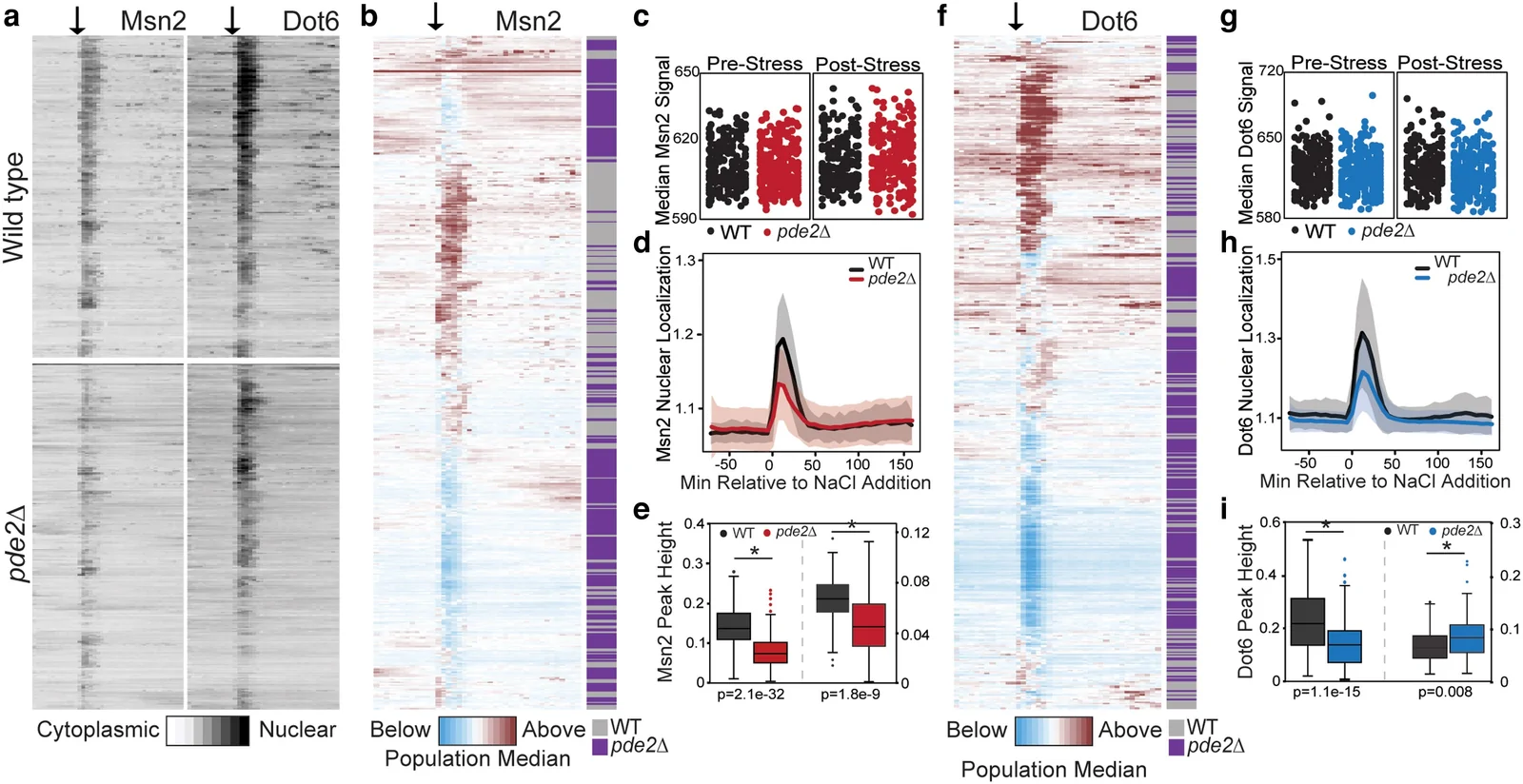
Pde2 regulates Msn2 nuclear translocation. Nuclear to cytoplasmic abundance of Msn2-mCherry or Dot6-GFP was measured for wild-type and *pde2Δ* cells mixed in the same chamber. a) Two panels showing the nuclear/cytoplasmic ratio for 212 wild-type-iRFP and 256 *pde2Δ* cells mixed in the same chamber over 5 biological replicates (strains shown separately in top and bottom panels for clarity), with Msn2 localization in the left column and Dot6 localization in the right column. Each row represents a different cell, and each column indicates a different time point spaced every 6 min. The time of NaCl addition is indicated by an arrow. b and f) Log_2_ change in nuclear/cytoplasmic ratio for b) Msn2-mCherry or f) Dot6-GFP normalized to the median value of each population at each time point, according to the key. Cells were organized separately in each panel by hierarchical clustering, and cell genotype was subsequently annotated in the single column to the right of panels b and f. Many *pde2Δ* cells (purple) fell into separate clusters based on Msn2-mCherry patterns (b), consistent with weaker nuclear translocation. c and g) Distribution of protein abundances (median signal in each cell) before and after stress. The gray line indicates the median. *P* values, Wilcoxon rank-sum test comparing wild-type and *pde2Δ* cells pre- and poststress. d) Median trace of cells from a), where shading represents 1 SD. e and h) Distributions of nuclear translocation peak heights as previously described (Kocik et al. 2026). The first pair of distributions on the left of each panel indicates when iRFP was expressed in wild-type cells; the second pair of distributions indicates when iRFP was expressed in *pde2Δ* cells (117 wild-type, 85 *pde2Δ* cells). Signal intensity differs between the pairs (shown on the left versus right axes) due to changes in lamp power over the span between these experiments. *P* values, Wilcoxon rank-sum test comparing wild-type and *pde2Δ* cells.

### Transcriptomic analysis

Data from Chasman et al. (2014) were analyzed in the context of gene sets identified in Kocik et al. (2026), focusing on the subset of clusters with expression changes in the wild type that are defective in *msn2Δmsn4Δ* cells. Transcription factor binding enriched for each cluster is available in Dataset EV2 (Kocik et al. 2026). The distribution of log_2_(fold change) values was taken for all ESR genes (Gasch et al. 2000). Distributions were compared using Wilcoxon rank-sum tests as indicated.

## Results and discussion

### Pde2 affects Msn2 activation dynamics

We previously developed a microfluidics system to follow nuclear translocation dynamics of genomically encoded Msn2-mCherry and Dot6-GFP expressed in the same cells (Bergen et al. 2022; Kocik et al. 2026). Here, we investigated the factors’ activation dynamics in a *pde2Δ* strain carrying both reporters. We mixed the mutant with a comparable wild-type strain containing a distinguishing iRFP marker, allowing mutant and wild type to be studied in the same microfluidics chamber. We measured Msn2-mCherry and Dot6-GFP nuclear translocation dynamics before and after exposure to an acute dose of 0.7 M sodium chloride (NaCl) inducing osmotic and ionic stress (Fig. 1a).

Cells lacking *PDE2* had a clear defect in Msn2 regulation after salt stress. Relative to wild type, *pde2Δ* cells showed normal Msn2-mCherry abundance (Fig. 1b and c) but significantly weaker nuclear translocation during NaCl stress (Fig. 1d and e). The duration of nuclear Msn2-mCherry was also truncated, implicating Pde2 in maintaining nuclear Msn2 localization (Fig. 1d). The defects in activation and nuclear retention were independent of which strain expressed the iRFP marker (Fig. 1e), confirming that Pde2 influences both the magnitude and timing of Msn2 activation.

The effect of *pde2Δ* on Dot6 was minimal compared to controls. Past work showed that the *msn2Δmsn4Δ* mutant harbors less Dot6-GFP protein, which we proposed is in part through direct Msn2/4 binding of the *DOT6* promoter (Kocik et al. 2026). Here, *pde2Δ* cells showed no difference in Dot6-GFP abundance (Fig. 1f and g), suggesting that cells either harbor enough Msn2/4 activity to maintain Dot6-GFP or that Msn2/4 influence Dot6 abundance through a Pde2-independent mechanism. The effect on Dot6-GFP activity was confounded by our system, since controls show that wild-type cells with the iRFP nuclear marker artifactually increased GFP nuclear signal over wild-type cells without the marker. The *pde2Δ* mutant showed Dot6-GFP translocation dynamics similar to the wild-type controls (Supplementary Fig. 1). Furthermore, the apparent difference in *pde2Δ* nuclear Dot6-GFP signal was lost when the iRFP marker was expressed in *pde2Δ* cells (Fig. 1h). Thus, despite limitations of our system, any Pde2-dependent effect on Dot6-GFP behavior is mild at best.

### Pde2 influences both iESR induction and rESR repression

We recently defined multiple sets of Msn2/4 targets based on temporal responses of wild-type, *msn2Δmsn4Δ*, and *dot6Δtod6Δ* cells responding to 0.7 M NaCl (Kocik et al. 2026). Here, we investigated the genes’ salt-responsive dependence on Pde2 versus Msn2/4 using published transcriptomic data from our lab (Chasman et al. 2014). Induced genes in clusters a to c (Fig. 2a) showed very similar expression defects in *pde2Δ* and *msn2Δmsn4Δ* cells studied in the same experiment (Fig. 2b). All 3 clusters are enriched for iESR genes, and direct assessment of the 283 iESR genes confirmed similar dependencies on both sets of regulators (Fig. 2c). Since *pde2Δ* cells show a major defect in Msn2 activation, the simplest explanation is that the Pde2 effect is primarily through Msn2/4, although it is possible that other regulators that control subsets of iESR genes could also be influenced by Pde2. In contrast, repressed genes influenced by Msn2/4 showed greater dependence on Pde2 in side-by-side experiments (Fig. 2b). This includes genes in clusters d and e that are enriched for RiBi genes and clusters f and g enriched for RP genes (Kocik et al. 2026). Furthermore, direct interrogation of those gene groups shows that the *pde2Δ* defect is significantly greater than the *msn2Δmsn4Δ* defect (*P* < 8e-5; Fig. 2c). This strongly suggests that Pde2 influences other regulators of rESR genes. One possibility is a direct connection to Dot6/Tod6: Although we could not confirm a *pde2Δ* defect in Dot6 activation due to fluorescent microscopy limitations, Dot6 can be regulated by PKA, raising the possibility of a direct connection (Deminoff et al. 2006; Lippman and Broach 2009; Kunkel et al. 2019). However, we returned to previous data from our lab that identified proteins immunoprecipitated with Pde2, which included the RP regulator Rap1 and the RiBi regulator Abf1 (MacGilvray et al. 2018). The functional importance of these interactions, observed both before and after salt stress, is unknown; however, the interactions elevate these regulators as candidates through which Pde2 may impact rESR expression.

**Fig. 2.**
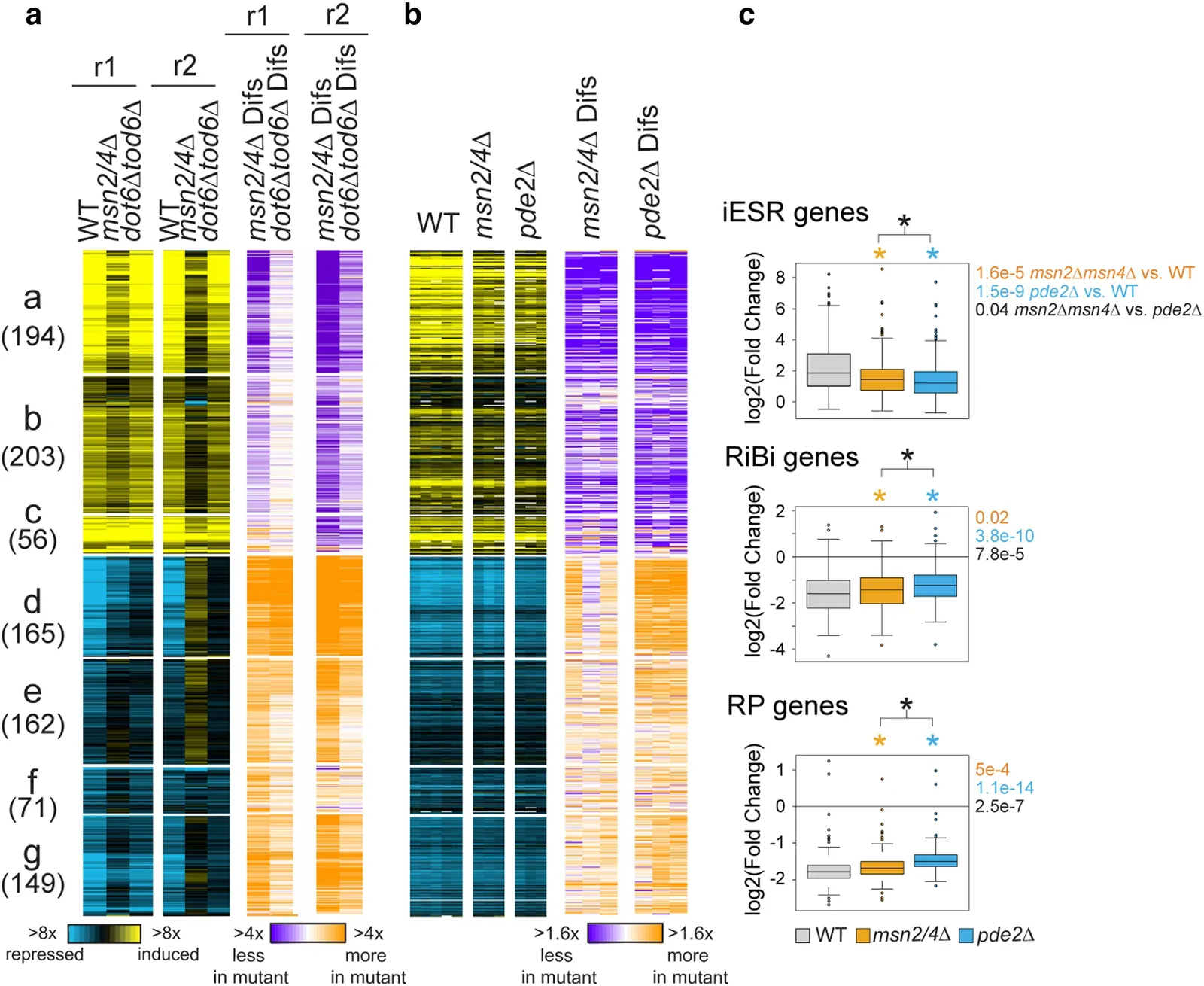
Contribution of Pde2 versus Msn2/4 to NaCl-responsive expression. Shown are 966 genes (rows) previously identified as induced or repressed in wild-type cells but with a defect in *msn2Δmsn4Δ* cells, as defined in Kocik et al. (2026). The figure shows seven clusters and four panels, showing expression in each strain from replicate r1 and r2. The number of genes per cluster is indicated in parentheses. a) Log_2_(fold change) in expression in wild type, *msn2Δmsn4Δ*, and *dot6Δtod6Δ* at 30 min after 0.7 M NaCl compared to unstressed cells from replicate time courses (*r*1, *r*2), according to the key (Kocik et al. 2026). Differences between mutant and wild type are shown according to the purple–orange key immediately below, for genes shown in a). b) As shown in a) but for WT (*n* = 5), *msn2Δmsn4Δ* (*n* = 3), and *pde2Δ* (*n* = 3) from Chasman et al. (2014). c) Distribution of log_2_(fold change) values for genes in the iESR (top), RiBi (middle), and RP (bottom) gene sets. Groups that were statistically different from wild type are indicated with an asterisk colored according to the key; groups different between *pde2Δ* and *msn2Δmsn4Δ* are indicated in black text. Asterisk, *P* < 0.05, Wilcoxon rank-sum test.

### Cells lacking PDE2 grow normally or faster after stress treatments

Decoupling the ESR has significant effects on poststress growth rate, but a quadruple mutant lacking *MSN2/MSN4/DOT6/TOD6* recovers with poststress growth rates that are equal to or better than wild type (Kocik et al. 2026). Since *pde2Δ* cells show a defect in both the iESR and rESR, we predicted poststress growth rates close to wild-type cells. Indeed, in 2 of 4 stresses tested (ethanol and basic pH), *pde2Δ* cells grew indistinguishably from wild type, whereas in response to low pH and salt studied here, *pde2Δ* cells grew slightly faster (Fig. 3). We also assessed cells lacking the other low-affinity phosphodiesterase Pde1, which is not known to play a role in stress response but does modulate cAMP levels. The single *pde1Δ* mutant did not have the same effect as *pde2Δ*, in some cases performing worse after stress treatments (Fig. 3). Cells lacking both Pde2 and Pde1 grew worse after most stresses and worse than either single-gene deletion, indicating that some cAMP phosphodiesterase activity is important for poststress fitness.

**Fig. 3.**
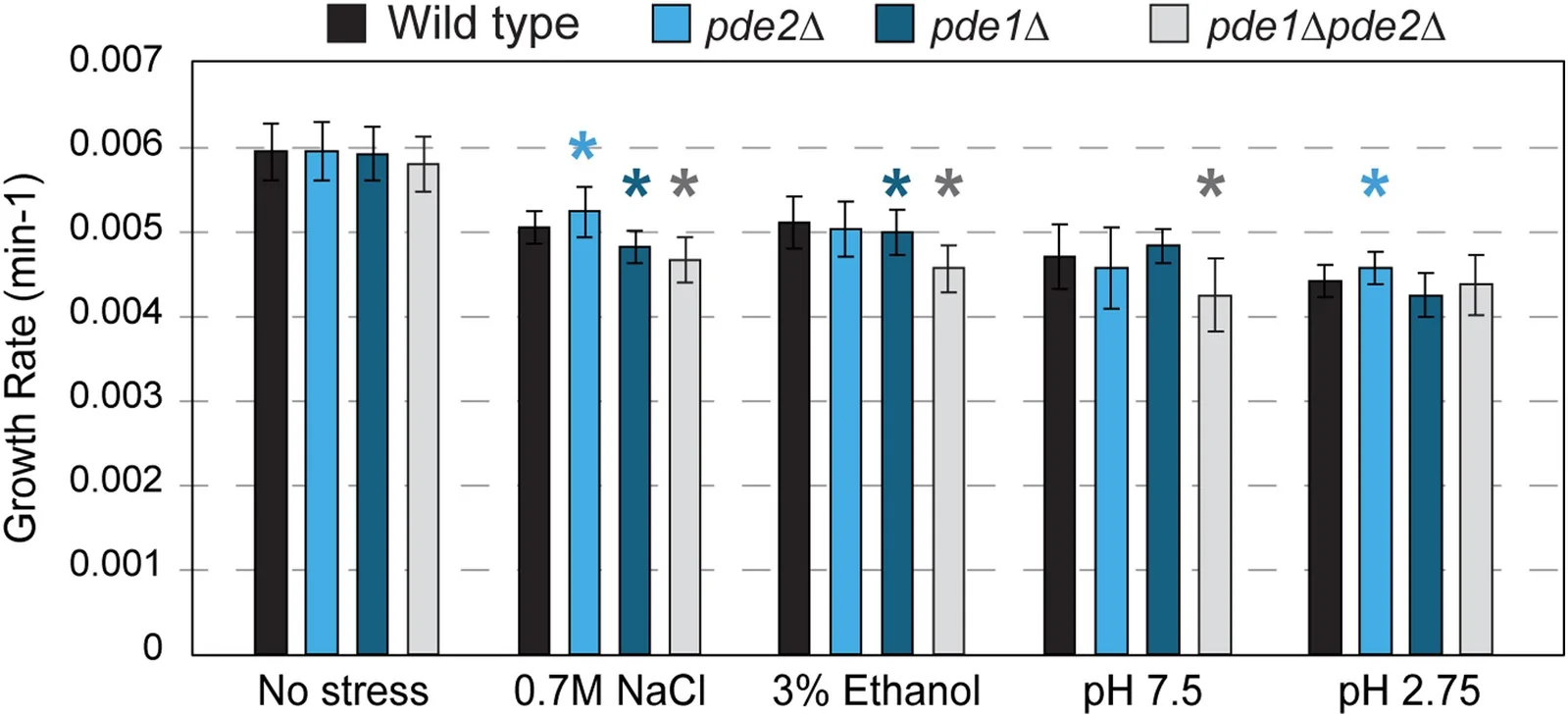
Pde2 is not required to maintain poststress growth rate. Average and standard deviation of growth rates calculated for four denoted strains growing in the absence of added stress (*n* = 12, far left) or after addition of NaCl (*n* = 7), ethanol (*n* = 7), or high or low pH (*n* = 5). Asterisk indicates *P* < 0.05, replicated paired T-tests.

### Concluding remarks

Overall, this work expands our understanding of ESR regulation and coordination, showing that Pde2 directly influences Msn2 activation dynamics and coordinates rESR repression through means beyond Msn2 input. This is consistent with the known inhibitory effect of PKA signaling on the ESR program (Klein and Struhl 1994; Smith et al. 1998; Garmendia-Torres et al. 2007; Lippman and Broach 2009; Kocik and Gasch 2022), but with other data suggest that Pde2 may play a more specific role than simply affecting PKA activity (MacGilvray et al. 2018). Several questions remain to be answered. How Pde2 is regulated during stress is not known, although it does undergo nucleocytoplasmic shuttling in a way that responds to PKA activity (Hu et al. 2010). Another question is if the physical interaction between Pde2 and PKA-regulated transcription factors, including Msn2, Abf1, and Rap1 discussed here, is important for transcriptional regulation. One possibility is that physical interactions and co-localized nuclear translocation are important for nuclear retention of Msn2, since the residual Msn2 nuclear localization in *pde2Δ* cells appeared truncated in response to salt stress. Future studies will be required to dissect these possibilities.

## Supplementary Material

jkag200_Supplementary_Data

## Data Availability

Strains are available upon request. Dataset S1 contains all microscopy data. Dataset S2 contains all growth curve data. Transcriptomic analyses used in this publication are from the NIH GEO database and assigned the identifiers GSE283327 (Kocik et al. 2026) and GSE60613 (Chasman et al. 2014). Supplemental material available at *G3* online.

## References

[jkag200-B1] Beck T, Hall MN. 1999. The TOR signalling pathway controls nuclear localization of nutrient-regulated transcription factors. Nature. 402:689–692. 10.1038/45287. 10604478

[jkag200-B2] Bergen AC, Kocik RA, Hose J, McClean MN, Gasch AP. 2022. Modeling single-cell phenotypes links yeast stress acclimation to transcriptional repression and pre-stress cellular states. eLife. 11:. 10.7554/eLife.82017. PMC9678356 36350693

[jkag200-B3] Berry DB et al 2011. Multiple means to the same end: the genetic basis of acquired stress resistance in yeast. PLoS Genet. 7:e1002353. 10.1371/journal.pgen.1002353. PMC3213159 22102822

[jkag200-B4] Berry DB, Gasch AP. 2008. Stress-activated genomic expression changes serve a preparative role for impending stress in yeast. Mol Biol Cell. 19:4580–4587. 10.1091/mbc.e07-07-0680. PMC2575158 18753408

[jkag200-B5] Chasman D et al 2014. Pathway connectivity and signaling coordination in the yeast stress-activated signaling network. Mol Syst Biol. 10:759. 10.15252/msb.20145120. PMC4299600 25411400

[jkag200-B6] Deminoff SJ, Howard SC, Hester A, Warner S, Herman PK. 2006. Using substrate-binding variants of the cAMP-dependent protein kinase to identify novel targets and a kinase domain important for substrate interactions in Saccharomyces cerevisiae. Genetics. 173:1909–1917. 10.1534/genetics.106.059238. PMC1569720 16751660

[jkag200-B7] Eisen MB, Spellman PT, Brown PO, Botstein D. 1998. Cluster analysis and display of genome-wide expression patterns. Proc Natl Acad Sci U S A. 95:14863–14868. 10.1073/pnas.95.25.14863. PMC24541 9843981

[jkag200-B8] Garmendia-Torres C, Goldbeter A, Jacquet M. 2007. Nucleocytoplasmic oscillations of the yeast transcription factor Msn2: evidence for periodic PKA activation. Curr Biol. 17:1044–1049. 10.1016/j.cub.2007.05.032. 17570669

[jkag200-B9] Garreau H et al 2000. Hyperphosphorylation of Msn2p and Msn4p in response to heat shock and the diauxic shift is inhibited by cAMP in Saccharomyces cerevisiae. Microbiology (Reading). 146:2113–2120. 10.1099/00221287-146-9-2113. 10974099

[jkag200-B10] Gasch AP et al 2000. Genomic expression programs in the response of yeast cells to environmental changes. Mol Biol Cell. 11:4241–4257. 10.1091/mbc.11.12.4241. PMC15070 11102521

[jkag200-B11] Guan Q, Haroon S, Bravo DG, Will JL, Gasch AP. 2012. Cellular memory of acquired stress resistance in Saccharomyces cerevisiae. Genetics. 192:495–505. 10.1534/genetics.112.143016. PMC3454879 22851651

[jkag200-B12] Ho Y-H, Gasch AP. 2015. Exploiting the yeast stress-activated signaling network to inform on stress biology and disease signaling. Curr Genet. 61:503–511. 10.1007/s00294-015-0491-0. PMC4800808 25957506

[jkag200-B13] Ho Y-H, Shishkova E, Hose J, Coon JJ, Gasch AP. 2018. Decoupling yeast cell division and stress defense implicates mRNA repression in translational reallocation during stress. Curr Biol. 28:2673–2680.e4. 10.1016/j.cub.2018.06.044. PMC6132260 30078561

[jkag200-B14] Hu Y, Liu E, Bai X, Zhang A. 2010. The localization and concentration of the *PDE2*-encoded high-affinity cAMP phosphodiesterase is regulated by cAMP-dependent protein kinase A in the yeast *Saccharomyces cerevisiae*. FEMS Yeast Res. 10:177–187. 10.1111/j.1567-1364.2009.00598.x. 20059552

[jkag200-B15] Huber A et al 2011. Sch9 regulates ribosome biogenesis via Stb3, Dot6 and Tod6 and the histone deacetylase complex RPD3L. EMBO J. 30:3052–3064. 10.1038/emboj.2011.221. PMC3160192 21730963

[jkag200-B16] Klein C, Struhl K. 1994. Protein kinase A mediates growth-regulated expression of yeast ribosomal protein genes by modulating RAP1 transcriptional activity. Mol Cell Biol. 14:1920–1928. 10.1128/mcb.14.3.1920-1928.1994. PMC358550 8114723

[jkag200-B17] Kocik RA, Cytrynbaum EG, Ahrens JM, McClean MN, Gasch AP. 2026. The importance of regulated resource reallocation during dynamic environmental shifts in yeast. EMBO J. 45:2808–2830. 10.1038/s44318-026-00727-x. PMC13084002 41813847

[jkag200-B18] Kocik RA, Gasch AP. 2022. Breadth and specificity in pleiotropic protein kinase A activity and environmental responses. Front Cell Dev Biol. 10:803392. 10.3389/fcell.2022.803392. PMC8888911 35252178

[jkag200-B19] Kunkel J, Luo X, Capaldi AP. 2019. Integrated TORC1 and PKA signaling control the temporal activation of glucose-induced gene expression in yeast. Nat Commun. 10:3558. 10.1038/s41467-019-11540-y. PMC6687784 31395866

[jkag200-B20] Lippman SI, Broach JR. 2009. Protein kinase A and TORC1 activate genes for ribosomal biogenesis by inactivating repressors encoded by Dot6 and its homolog Tod6. Proc Natl Acad Sci U S A. 106:19928–19933. 10.1073/pnas.0907027106. PMC2775034 19901341

[jkag200-B21] Ma P, Wera S, Van Dijck P, Thevelein JM. 1999. The PDE1-encoded low-affinity phosphodiesterase in the yeast Saccharomyces cerevisiae has a specific function in controlling agonist-induced cAMP signaling. Mol Biol Cell. 10:91–104. 10.1091/mbc.10.1.91. PMC25156 9880329

[jkag200-B22] MacGilvray ME et al 2018. Network inference reveals novel connections in pathways regulating growth and defense in the yeast salt response. PLoS Comput Biol. 13:e1006088. 10.1371/journal.pcbi.1006088. PMC5940180 29738528

[jkag200-B23] Nikawa J, Sass P, Wigler M. 1987. Cloning and characterization of the low-affinity cyclic AMP phosphodiesterase gene of Saccharomyces cerevisiae. Mol Cell Biol. 7:3629–3636. 10.1128/mcb.7.10.3629-3636.1987. PMC368017 2824992

[jkag200-B24] Saldanha AJ . 2004. Java Treeview—extensible visualization of microarray data. Bioinformatics. 20:3246–3248. 10.1093/bioinformatics/bth349. 15180930

[jkag200-B25] Smith A, Ward MP, Garrett S. 1998. Yeast PKA represses Msn2p/Msn4p-dependent gene expression to regulate growth, stress response and glycogen accumulation. EMBO J. 17:3556–3564. 10.1093/emboj/17.13.3556. PMC1170692 9649426

