# Supplementary material for: Investigating the role of phosphodiesterase Pde2 in coordinating resource reallocation through the environmental stress response
Source: G3 (Bethesda). 2026 Sep 22;16(11):jkag200. doi: 10.1093/g3journal/jkag200 (PMC13732475; doi:10.1093/g3journal/jkag200)
Supplement: Supplementary file 1 [file jkag200_supplementary_data.zip › Figure_S1_G3-2026-407062.pdf]

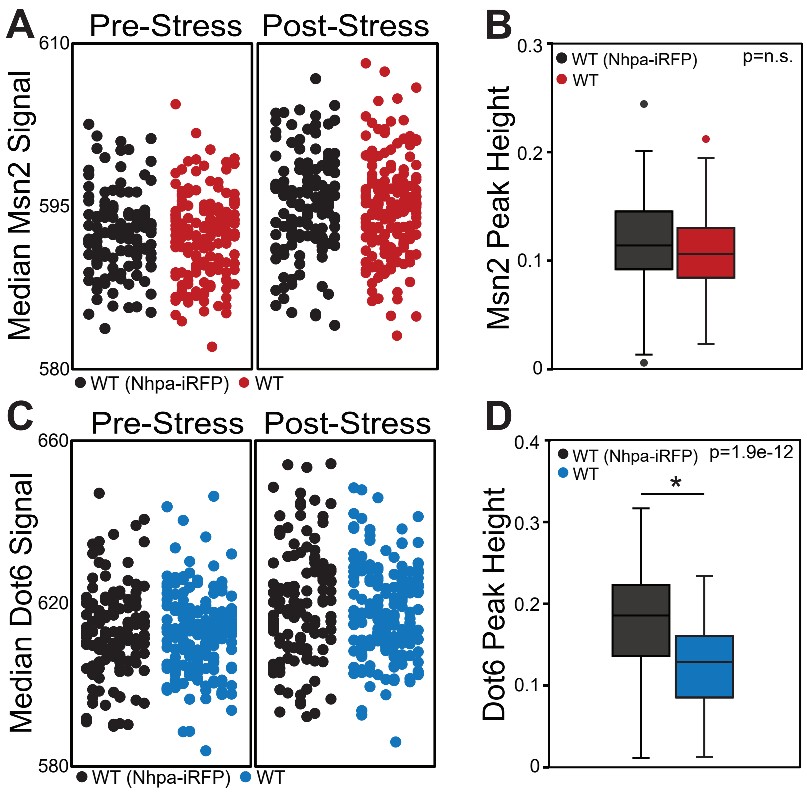

Figure S1. iRFP expression can affect peak height measurement in the GFP channel. Control experiments mixed two wild-type strains, both expressing Msn2-mCherry and Dot6-GFP but where only one strain carried the additional iRFP marker. **A and C**) Median pixel intensity of Msn2-mCherry (A) or Dot6-GFP (C). Grey line indicates median. P-values, Wilcoxon rank-sum test comparing wild-type (with Nhp6a-iRFP nuclear marker) and wild-type (without Nhp6a-iRFP) cells pre- and post-stress. **B and D**) Nuclear translocation peak heights of Msn2-mCherry (B) and Dot6-GFP (D) are shown for the two strains. Cells carrying the iRFP marker reproducibly showed a larger apparent nuclear translocation signal in the GFP channel (see **D**), indicating an artifact of the iRFP marker. P-values based on Wilcoxon rank-sum test.
